# Enhanced Hippocampus–Nidopallium Caudolaterale Interaction in Visual–Spatial Associative Learning of Pigeons

**DOI:** 10.3390/ani14030456

**Published:** 2024-01-30

**Authors:** Jun-Yao Zhu, Zhi-Heng Zhang, Gang Liu, Hong Wan

**Affiliations:** 1 School of Electrical and Information Engineering, Zhengzhou University, Zhengzhou 450001, China; zhujunyao000@163.com (J.-Y.Z.); zhangzhiheng_zzu@163.com (Z.-H.Z.); 2 Henan Key Laboratory of Brain Science and Brain-Computer Interface Technology, Zhengzhou 450001, China; 3 Shanghai Key Laboratory of Brain-Machine Intelligence for Information Behavior, Shanghai International Studies University, Shanghai 201613, China

**Keywords:** spatial associative learning, hippocampus–nidopallium caudolaterale information flow, phase transfer entropy, pigeon local field potential

## Abstract

**Simple Summary:**

Birds have excellent visual–spatial associative learning abilities and heavily rely on them for navigation, homing, and foraging. In birds, the hippocampus (Hp) and nidopallium caudolaterale (NCL) play critical roles in this cognitive process. To enhance our understanding of how bird Hp and NCL cooperate to support visual–spatial associative learning, we examined the neural activities of the Hp and NCL of pigeons and explored their interaction dynamics during the learning process. We found that pigeons’ behavioral changes during learning are accompanied by modifications in their interaction patterns in the Hp–NCL network. The enhanced theta-band synchronization between the Hp and NCL, as well as the dynamics of Hp–NCL interaction, provides insight into the potential mechanism of the Hp–NCL network during spatial associative learning.

**Abstract:**

Learning the spatial location associated with visual cues in the environment is crucial for survival. This ability is supported by a distributed interactive network. However, it is not fully understood how the most important task-related brain areas in birds, the hippocampus (Hp) and the nidopallium caudolaterale (NCL), interact in visual–spatial associative learning. To investigate the mechanisms of such coordination, synchrony and causal analysis were applied to the local field potentials of the Hp and NCL of pigeons while performing a visual–spatial associative learning task. The results showed that, over the course of learning, theta-band (4–12 Hz) oscillations in the Hp and NCL became strongly synchronized before the pigeons entered the critical choice platform for turning, with the information flowing preferentially from the Hp to the NCL. The learning process was primarily associated with the increased Hp–NCL interaction of theta rhythm. Meanwhile, the enhanced theta-band Hp–NCL interaction predicted the correct choice, supporting the pigeons’ use of visual cues to guide navigation. These findings provide insight into the dynamics of Hp–NCL interaction during visual–spatial associative learning, serving to reveal the mechanisms of Hp and NCL coordination during the encoding and retrieval of visual–spatial associative memory.

## 1. Introduction

Visual–spatial associative learning refers to the ability to link visual cues and spatial locations together in memory [1,2], where animals adjust their navigational patterns to goal locations based on previously presented visual cues in the environment. This is a key aspect of cognition and is crucial for the survival of animals. However, the neural mechanisms underlying visual–spatial associative learning are still under debate.

Accumulating evidence shows that a distributed brain network comprising the primary sensory areas, the hippocampus (Hp), and higher-order cortical areas involved in executive function supports animals in realizing this complex cognitive process [1,3,4]. The interplay between the Hp and the prefrontal cortex (PFC) plays a key role in associative learning in mammals [5,6]. As the most important task-related brain area, the Hp receives highly processed visual and spatial information from different cortical streams and integrates them to form visual–spatial associative memory [7,8,9]. The PFC participates in associative memory retrieval and synthesizes visual cues and mnemonic information to direct behavior appropriately [10,11]. It is generally believed that these two brain areas interact and cooperate to share context (sensory or spatial), control, and mnemonic information through temporal-specific neural oscillations to support the formation of associations and flexible decision-making behavior in associative tasks [12,13].

Avians rely heavily on visual cues for navigation and foraging [14]. The bird Hp is essential for regulating the spatial map-like representations of visual features, and it associates visual cues and spatial locations to guide homing behavior [15,16,17,18]. The bird Hp and mammalian Hp have the same origin [19]. After close to 310 million years of independent evolution, although there are some differences in their structure, and the correspondence between their subdivisions is still under debate, researchers agree that the bird Hp shows homology with that of mammals [20,21,22]. Like the mammalian system, the bird Hp has access to highly processed visual information through the parahippocampal area (APH) [8,19], which provides the neural circuit basis for the formation of visual–spatial associative memory. Functionally, many features of hippocampal functions are conserved in the bird Hp, especially those used for spatial-related learning and memory tasks [23,24,25]. Lesion research indicates that damage to the Hp of pigeons impairs their visual–spatial associative learning [26]. In terms of neural activity, neurons in the Hp encode spatial information guided by visual cues, and the enhanced theta-band activity in the Hp is associated with spatial learning [27,28]. Collectively, these findings suggest that the bird Hp is critical in associating visual cues with spatial locations and may be involved in the formation of visual–spatial associative memories during the learning process. An increasing number of studies are indicating that the bird nidopallium caudolaterale (NCL) and the mammalian PFC are similar structures involved in cognitive processes [29,30]. In associative learning, the neuronal firing of the NCL becomes selective for associations, supporting the notion that like the mammalian PFC, the bird NCL participates in associative memory retrieval and synthesizes the external sensory cues and internal mnemonic information to support flexible decision making [31,32]. The bird Hp and NCL play complementary roles in associative learning. However, it is unclear how the two brain areas cooperate to support the animals in learning to use visual cues to guide their navigational behavior.

Coordinated activity between the Hp and NCL of birds requires an anatomical connection. Shanahan et al. [33] presented a large-scale “wiring diagram” for the forebrain of a bird, according to which there are multiple indirect connections between the bird Hp and NCL. Recent electrophysiological studies on pigeons have also demonstrated the existence of information pathways between the Hp and NCL [28,34]. A study about pigeons performing a goal-directed decision-making task found that there was an information flow from the Hp to the NCL whilst pigeons were in the turning area of a T-maze [34]. In another study, researchers demonstrated that enhanced Hp–NCL connectivity was associated with the formation of stable routes in pigeons during spatial learning [28]. However, it is unknown whether there is an interaction between these two brain areas in visual–spatial associative learning. If there is, how does the interaction evolve during the learning process?

Therefore, in this study, to investigate the dynamics of Hp–NCL interactions in pigeons during visual–spatial associative learning, synchrony and causal analysis were applied to local field potentials (LFPs) recorded from the Hp and NCL of pigeons performing a visual–spatial associative learning task. According to the functions of the bird Hp and NCL, we hypothesize that there may be an information interaction between the Hp and NCL during the learning procedure, and the interaction strength may evolve as the animals learn to use visual cues to guide their navigational behavior.

## 2. Materials and Methods

### 2.1. Subjects

A total of six pigeons (*Columba livia*) weighing 400 to 500 g were used in this study. After surgery, they were housed individually in iron cages (80 × 60 × 60 cm) with plenty of sunlight, good ventilation, and free access to water and food. The pigeons recovered from surgery for at least one week before the visual–spatial associative learning task. During learning, the pigeons were placed on a food deprivation regiment that kept them at 80–90% of their free-feeding body weight. The animals were kept on a 12-h light/12-h dark schedule. All experiments were performed from 15 to 17 o’clock. All experimental procedures involving animal surgery were approved by the Life Science Ethical Review Committee of Zhengzhou University. Best efforts were made to optimize welfare.

### 2.2. Surgical Implantation of Electrodes and Data Acquisition

**Figure 1:**
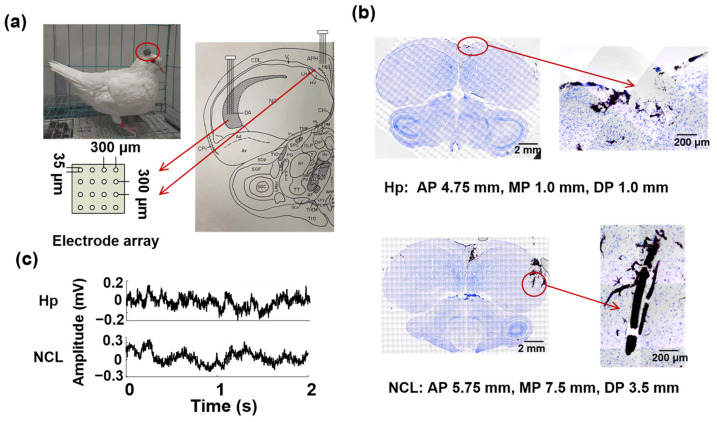
Electrode implantation and data acquisition. (**a**) Left: Photograph of a pigeon (P1) implanted with microelectrode arrays. Right: Two 16-channel microelectrode arrays were implanted in the hippocampus (Hp) and nidopallium caudolaterale (NCL); (**b**) Histological verifications of recording sites. Representative examples of electrode positions recorded in the Hp (up) and NCL (down) within the same animal (P1) are presented. AP: anteroposterior, MP: mediolateral, DP: dorsoventral. (**c**) Examples of simultaneous local field potential signals (LFPs) recorded from Hp and NCL.

The pigeons were operated on under pentobarbital sodium anesthesia at a concentration of 1.5%. The pentobarbital sodium was injected into the animal’s body through intramuscular injection in the chest. The pigeon head was fixed on a stereotaxic apparatus, then two 16-channel recording microelectrode arrays (4 × 4 arrays, Hong Kong Plexon Inc., Hong Kong, China, Figure 1a) were chronically implanted in the left Hp (AP 4.5 mm; ML 1.0 mm; DV 0.5 to 1.5 mm) and NCL (AP 5.5 mm; ML 7.5 mm; DV 2.5 to 3.5 mm, Figure 1b). The coordinates were obtained from the Karten and Hodos stereotaxic atlas of the pigeon brain [35]. A 128-channel Cerebus TM Multichannel Acquisition Processor (Blackrock Microsystems, Salt Lake City, UT, USA) was used to record LFPs from the Hp and NCL of pigeons. The sampling rate was 2 kHz and LFPs were filtered by a 0–250 Hz Butterworth low-pass filter.

### 2.3. Behavioural Task

**Figure 2:**
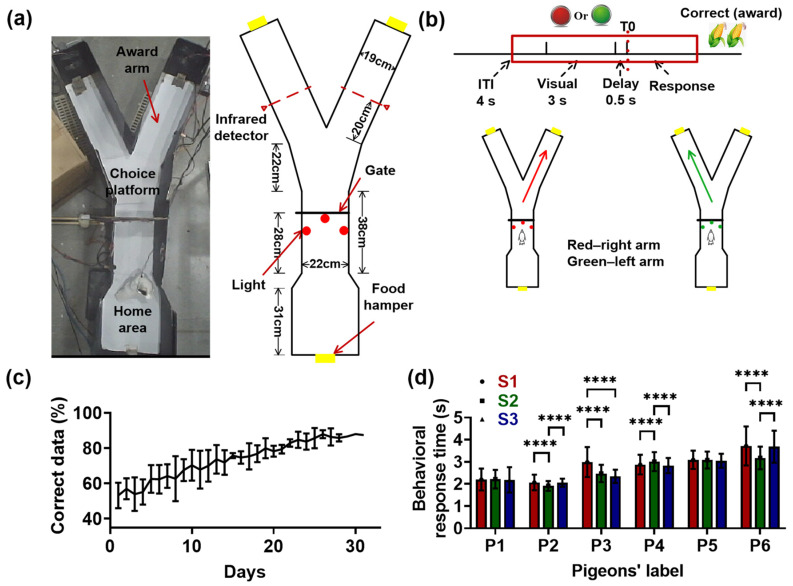
Visual–spatial associative learning paradigm and the behavioral performance of pigeons. (**a**) Schematic illustration of the visual–spatial associative learning in the “Y” maze. Animals initiated each trial by experiencing an inter-trial interval (ITI) in the home area for about 4 s, and then the visual cue (red or green light; color pseudo-random) was presented for 3 s. This was followed by a delay interval of 0.5 s, in which the visual cue disappeared. The trials ended with the animal responding to one of the two arms, depending on the light color. The red light corresponded to the right arm and the green light was associated with the left arm. The infrared detectors recorded the moments when the pigeons completed the turning and reached the target arm. T0 indicates the end of the delay period, when the gate begins to open. (**b**) Diagram of the visual–spatial associative learning. The red block represents the epochs to be analyzed: the inter-trial interval (ITI) of 1.5 s, visual identification of 3 s, delay period of 0.5 s, and tuning period of 3.0 s. The right choices were rewarded with grain for 4 s. (**c**) The accurate rate of pigeons plotted as a function of days. Data are presented as mean ± std. (**d**) The behavioral response time (defined as the time it took for a pigeon to reach the target arm from the moment the door began to open) of pigeons during learning (**** p<0.0001; Friedman ANOVA). Data are presented as mean ± std. S1 represents learning stage 1, and so forth.

Pre-training: To reduce the time cost of animals learning an associative task, pre-training was conducted on the pigeons in the Y maze before surgery (Figure 2a), familiarizing the animals with the experimental process (starting from the home area, reaching the arms, and then returning home). Therefore, in pre-training, the pigeons were placed at the starting position (the home area). About 4 s later, the gate began to open, and the animals started to explore the maze. When the animals reached one of the two arms, the food hamper at the end of the arm popped up automatically. After the animals enjoyed a 4 s food reward, the food hamper in the home area was opened to guide the pigeons back to the home area. After the pigeons returned home, the gate and the food hamper were closed. The pigeons were waiting in the home area for the next trial. The duration of pre-training and the number of trials for each pigeon differed. Some pigeons could become familiar with the maze and easily find food within 3–4 days. The pre-training for some pigeons lasted for 6–7 days. Experimenters assessed whether pre-training could be terminated by recording the behavior of pigeons in the maze through a camera. Pigeons that did not explore the maze for 4–5 consecutive days were excluded. After the pigeons had completed pre-training proficiently, the electrode implantation operation was performed on them.

Visual–spatial associative learning task: Six pigeons in this experiment recovered from surgery within a week. After a 7-day recovery period, the pigeons began to learn the visual–spatial associative task. Figure 2a,b shows the schematic of the associative learning task. First, the pigeons experienced a 4 s inter-trial interval (ITI) in the home area. Next, the lights (red or green, color pseudo-random) in the home area were turned on, followed by a delay. The light duration was 3 s and the delay period was 500 ms. Then, the gate began to open and the pigeons started to run towards either of the two arms, depending on their visual identity. The red light was associated with the right arm, while the green light corresponded to the left arm. Correct choices were rewarded with 4 s of grain delivered via the food hamper. After the pigeons enjoyed the food reward, the food hamper was closed and the animals returned to the home area, waiting for the next trial. The training was terminated when the pigeons reached the criterion of an 85% correct rate for three consecutive sessions. The correct rate was obtained by dividing the number of correct trials in a session by the total number of trials in that session. Video clips of pigeons’ visual–spatial associative learning and detailed behavioral data are provided in the Supplementary Materials (Video S1, Table S1).

In all behavioral experiments, the infrared detectors in the maze recorded the moments when the pigeons completed the turning and reached the target arm, and there was a camera was positioned directly above the maze to record the behavior of pigeons in the maze.

### 2.4. LFPs Processing and Analysis

Data processing was performed before analysis. First, only trials with a pigeon behavioral response time of less than 5 s were reserved. The behavioral response time was defined as the time it took for a pigeon to reach the target arm from the moment the door began to open. The trials containing strong motion artifacts were also removed. Then, common average referencing was applied for the Hp and NCL channels of the remaining trials to remove the spatially correlated noise [36]. Finally, the LFPs from the last 1.5 s of ITI to the 3 s after the gate began to open (a total of 8 s) were used for the subsequent analysis.

(1) Phase locking value (*PLV*)

*PLV* measures the consistency of phase differences for the LFP pairs across the repeated trials, independent of the absolute phases and amplitudes [37]. To measure the synchronous activity of Hp and NCL, *PLV* was applied for the LFPs recorded from them. First, LFPs were transformed to the time-frequency domain using complex Morlet wavelets or a zero-phase lag finite impulse response (FIR) filter, from which the phase time series for each frequency band was extracted. Then, *PLV* was used for the phase time series pairs across the repeated trials.

(1) $${PLV}_{p,\;q}(t,\;f)=\left|\frac{1}{nTrials}\sum_{trl=1}^{nTrials}exp(i[\varphi_{p,\;trl}(t,\;f)-\varphi_{q,\;trl}(t,\;f)])\right|$$

where p and q are the electrode numbers of the Hp and NCL, respectively. φ is the phase time series and nTrials is the total number of the repeated trials.

PLV was performed separately for each electrode pair (a total of 256 electrode pairs for two 16-channel electrode arrays) and normalized to a z-score-like statistic by subtracting the mean and dividing by the standard deviation of the *PLV*, calculated across 50 random permutations of $\varphi_{p,\;trl}(t,\;f)$ across trials [38].

(2) Phase transfer entropy (*PTE*)

To measure the directional influence between the Hp and NCL, *PTE* was applied to the LFPs recorded from brain areas. *PTE* is a description of Wiener–Granger causality using the framework of information theory, i.e., that a source signal has a causal influence on a target signal if knowing the past of both signals improves the ability to predict the target’s future compared with knowing only the target’s past [39]. *PTE* is considered to be a “non-linear version of Granger causality”, and it has been widely used to identify the directional interaction between brain areas [40,41]. For two variables X and Y, the *PTE* from X to Y can be defined as follows:

(2) $$PTE(X\to Y,f)=\sum_{\varphi_{t}^{y},\;\varphi_{t-1}^{y,\;dy},\;\varphi_{t-u}^{x,\;dx}}p\left(\varphi_{t}^{y},\;\varphi_{t-1}^{y,\;dy},\;\varphi_{t-u}^{x,\;dx}\right)log\frac{p\left(\varphi_{t}^{y}|\varphi_{t-1}^{y,\;dy},\;\varphi_{t-u}^{x,\;dx}\right)}{p\left(\varphi_{t}^{y}|\varphi_{t-1}^{y,\;dy}\right)}$$

where $\varphi_{t}^{x}$ and $\varphi_{t}^{y}$ are the instantaneous phase time series of $x_{t}$ and $y_{t}$ at frequency *f*; $\varphi_{t-1}^{y,\;dy}$ and, $\varphi_{t-u}^{x,\;dx}$ $\in R^{D\times d}$ are the time-embedded series of $\varphi_{t}^{y}$ and $\varphi_{t}^{x}$; D=T−(τ×(d−1))−u, in which τ and, d ∈N are the embedding dimension and embedding delay, respectively. T indicates the length of $\varphi_{t}^{y}$, and u ∈N represents the interaction lag between X and Y. p is the probability distribution (PDF).

To improve the accuracy of *PTE* in identifying the interaction strength and direction, *PTE* is extended to the ensemble method, where the independent repetition trials of an experimental condition are taken as an ensemble of realizations, and various PDFs are estimated from the ensemble members. *PTE* can be rewritten as follows:

(3) $$PTE\left(X\;\to Y,f\right)=\sum_{\varphi_{t}^{y}\left(r\right),\;\varphi_{t-1}^{y,\;dy}\left(r\right),\;\varphi_{t-u}^{x,\;dx}\left(r\right)}p\left(\varphi_{t}^{y}\left(r\right),\;\varphi_{t-1}^{y,\;dy}\left(r\right),\;\varphi_{t-u}^{x,\;dx}\left(r\right)\right)\times log\frac{p\left(\varphi_{t}^{y}(r)|\varphi_{t-1}^{y,\;dy}(r),\;\varphi_{t-u}^{x,\;dx}(r)\right)}{p\left(\varphi_{t}^{y}(r)|\varphi_{t-1}^{y,\;dy}(r)\right)}$$

where r is the number of independent repetition trials.

*PTE* is a biased estimate, and it may still be non-zero even in the absence of an interaction between X and Y. To reduce bias, the differential PTE is used:

(4) $$dPTE\left(X\;\to \;Y,\;f\right)=PTE\left(X\;\to \;Y,\;f\right)-PTE(Y\;\to \;X,\;f)$$

dPTE(X → Y, f)> 0 indicates that the information flow is preferentially from X to Y, and dPTE(X → Y, f)< 0 defines the reverse direction. In the case with no preferential direction of interaction, $dPTE\left(X\to Y,f\right)$ = 0.

To identify the interaction lag δ between X and Y, the scanning method is applied to estimate δ; when $dPTE\left(X\to Y,f,u\right)$ is maximal, u is equal to δ.

(5) $$\delta ={argmax}_{u}(dPTE\left(X\to Y,f,u\right))$$

To test the statistical significance of $dPTE\left(X\;\to \;Y,\;f\right)$, the source variable X is time-shuffled to generate the surrogate data, and the $dPTE\left(X\;\to \;Y,\;f\right)$ for 200 sets of surrogate data is calculated to construct the null hypothesis distribution. The null hypothesis of raw data can be rejected or retained by comparing the $dPTE\left(X\;\to \;Y,\;f\right)$ of the raw data to the null hypothesis distribution at a significance level of 5%. 

Here, *PTE* was used to detect the interaction between the Hp and NCL. First, the delta (1–3 Hz), theta (4–12 Hz), beta (13–30 Hz), slow-gamma (31–45 Hz), and fast-gamma (55–80 Hz) bands of the LFPs recorded from the Hp and NCL were filtered by a zero-phase lag FIR filter with the order defined as 3 *r*, where *r* was the ratio of the sampling rate to the low-frequency cutoff of the filter, rounded down [42]. Then, the phase time series was extracted via Hilbert transform. Finally, a 0.4 s sliding window with 0.2 s step size divided the phase time series (a total of 8 s) into 39 overlapping bins, and PTE was applied to each bin. *dPTE* was normalized to a z-score-like statistic by subtracting the mean and dividing by the standard deviation of the *dPTE* values calculated by the surrogate data.

We assumed that all trials in a session had equivalent brain activity. Therefore, the correct trials in a session were considered repetition trials, and the multi-channel LFPs recorded from the Hp were pooled together, as well as the NCL LFPs. For each estimation, 200 LFPs, drawn randomly from the Hp and NCL, were combined into 200 signal pairs, and the interaction lag was searched in the range from 1 to 40 ms. The histogram-based method was used to estimate the PTE metric value, and the bin width was set as 1.8. This process was repeated 30 times. The significance of the *dPTE* value for each estimation was tested against the 200 surrogate data sets (p < 0.05) that were generated by time-shuffling the phase time series of Hp in each trial so that the predictability between the Hp and NCL was destroyed. Then, the binomial test was applied to establish the statistical significance of all repetitions. The robustness of *PTE* to the bin size has been analyzed in the Supplementary Materials (Figure S1, Table S2).

### 2.5. Statistical Analysis

Statistical analysis was performed with MATLAB and SPSS. Two-group comparisons with a normal distribution or large sample size were assessed by paired t-test. Otherwise, a non-parametric Wilcoxon signed-rank test was used. Multiple-group comparisons were assessed by Friedman analysis of variance (ANOVA), followed by a post hoc Bonferroni multiple comparison test. The analysis of the correlation between the pigeon’s behavioral performance and the PTE metric value was conducted using the two-sided Pearson’s correlation. Statistical results were presented as mean ± standard deviation (std), with the statistics including the Chi-square value for Friedman ANOVA, the value of the test statistic (tstat) for a paired t-test, the value of the normal statistic (zval) for a Wilcoxon signed-rank test, degrees of freedom (df), and p value. For tests that do not provide df, the sample size used for statistical testing has been reported. Differences were considered significant for p < 0.05. **** p < 0.0001, *** p < 0.001, ** p < 0.01, and * p < 0.05.

## 3. Results

### 3.1. Behavioural Performance of Pigeons during the Visual–Spatial Associative Learning

Six pigeons (numbered P1, P2, P3, P4, P5, and P6) were trained to learn the visual–spatial associative task. All pigeons began with a chance performance (from 40% to 60%), and after 20 training sessions, the percentage of correct trials reached 80% (Figure 2c, except for P4, with about 22 sessions being needed). Pigeons P1, P2, P3, P4, P5, and P6 terminated the visual–spatial associative learning task after 28, 26, 25, 31, 28, and 28 training sessions, respectively. Therefore, the learning process was divided into three stages: S1 (early stage, 1–10 sessions, 198 ± 12 trials for six pigeons), S2 (middle stage, 11–19 sessions, 182 ± 21 trials; 11–21 sessions for P4), and S3 (late stage, 20–end sessions, 180 ± 33 trials; 22–end sessions for P4). Almost all pigeons had an accuracy rate of over 80% in S3, indicating that they had learned the visual–spatial associative task. 

The behavioral response time (the time it took for a pigeon to reach the target arm from the moment the door began to open) of pigeons showed different trends during the learning process. The behavioral response times of P1 and P5 had no significant difference in S1, S2, and S3 (p > 0.05, Chi-square = 1.0502 for P1, 2.1477 for P5, df = 2; Friedman ANOVA). The behavioral response times of P2 and P6 showed a trend of first decreasing and then increasing, while that of P4 was exactly the opposite, first increasing and then decreasing (p < 0.0001, Chi-square = 40.9361 for P2, df = 2; p < 0.0001, Chi-square = 30.4798 for P4, df = 2; p < 0.0001, Chi-square = 74.5803 for P6, df = 2; Friedman ANOVA). Only the behavioral response time of P3 decreased with learning (p < 0.0001, Chi-square = 80.5019 for P3, df = 2; Friedman ANOVA).

### 3.2. The Interaction between the Hp and NCL of Pigeons in the Visual–Spatial Associative Task

**Figure 3:**
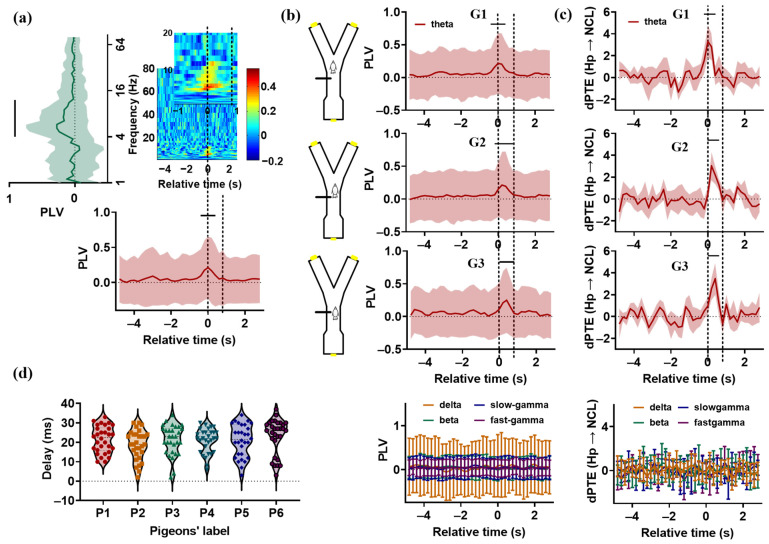
Synchronization and causal analysis of the Hp and NCL in pigeons’ visual–spatial associative task. (**a**) For P1, the mean synchrony (phase locking value (*PLV*), averaged across all electrode pairs) between the Hp and NCL is plotted as a spectrogram across the trial’s relative time and frequency. Time 0 (T0) indicates the end of the delay period, when the gate begins to open. The two dashed lines represent the beginning and full opening of the gate. It took about 0.8 s for the gate to be fully opened. Left: The *PLV* during the period from 0.4 s before T0 to 0.4 s after T0, plotted as a function of frequency (against the *PLV* during the ITI period for delta, theta, beta, slow-gamma, fast-gamma frequency bands; *p* > 0.05, tstat = 0.4430 for delta; *p* < 0.0001, tstat = 3.9999 for theta; *p* > 0.05, tstat = 0.2550 for beta; *p* > 0.05, tstat = 0.8138 for slow-gamma; *p* > 0.05, tstat = 0.60337 for fast-gamma; *df* = 224, paired *t*-test, electrode pairs = 225). “—” indicates significant difference. Bottom: The theta-band *PLV* plotted across the trial’s relative time (against the *PLV* during the ITI period for each time bin: *p* < 0.01, tstat = 2.7589 for the bin from 0.4 s before T0 to T0; *p* < 0.0001, tstat = 3.7141 for the bin from 0.2 s before T0 to 0.2 s after T0; *p* < 0.01, tstat = 2.6676 for the bin from T0 to 0.4 s after T0; *df* = 224, paired *t*-test). Data are presented as mean ± std. (**b**) Left: Schematic illustration of the position of the pigeons in the maze when the gate was fully opened. According to the video, the pigeons in G1 had already reached the choice platform when the gate was fully opened, while the pigeons in G2 and G3 were entering or preparing to enter the choice platform. Right: The *PLV* plotted across the trial’s relative time. Top right: The theta-band *PLV* for G1, G2, and G3. Bottom right: The *PLV* of the delta, beta, slow-gamma, and fast-gamma frequency bands for all pigeons. The two dashed lines represent the beginning and full opening of the gate. Data are presented as mean ± std. (**c**) The *dPTE* (Hp → NCL) plotted as a function of the trial’s relative time. Top: The *dPTE* (Hp → NCL) of theta-bands for G1, G2, and G3. Bottom: The *dPTE* (Hp → NCL) of the delta, beta, slow-gamma, and fast-gamma frequency bands for all pigeons. The two dashed lines represent the beginning and full opening of the gate. Data are presented as mean ± std. (**d**) The interaction lag values between the Hp and NCL for all pigeons were searched in the range from 0 to 40 ms.

We first examined the interaction between the Hp and NCL of pigeons when they had reached the 80% performance criterion in the visual–spatial associative tasks. The LFPs during the period from ITI to the pigoen’s completion of behavioral response in learning stage S3 were analyzed (the red block in Figure 2b), and the moment the gate began to open was taken as T0; that is, time 0 on the horizontal axis of the graphs in Figure 3.

To measure the synchronization between the Hp and NCL of pigeons in visual–spatial associative tasks, PLV was applied for the trials in which the pigeons had learned the task. We found that the oscillations the Hp and NCL were highly synchronized at the theta (from 4 to 12 Hz) frequency band for all pigeons (Figure 3a,b), and the enhanced Hp–NCL synchronization occurred at different task times (Figure S2). Based on this, we divided the pigeons into three groups: G1 (P1, P2), G2 (P3, P4, and P5), and G3 (P6). For the pigeons in G1, enhanced synchrony was observed during the period from 0.4 s before T0 to 0.4 s after T0. The statistical results are presented in Table 1 (the PLV of each time bin against that of the ITI period for delta, theta, beta, slow-gamma, and fast-gamma frequency bands. Electrode pairs = 225 and, 256 for P1 and P2, respectively). The synchronization reached the peak value at T0, with the animals beginning to run towards the choice platform. For G2 and G3, enhanced synchrony was observed during the period from 0.2 s before T0 to 0.8 s after T0, and from T0 to 0.8 s after T0, respectively (electrode pairs = 256, 225, 225, and 256 for P3, P4, P5, and P6, respectively). The peak values were noted at about 0.2 s and 0.4 s after T0 for G2 and G3, respectively. These results indicate that the theta-band oscillations in the Hp and NCL became highly synchronized before the animals entered the critical choice platform, reflecting the information interaction between these two brain areas.

To measure the directional influence between the Hp and NCL of pigeons during visual–spatial associative tasks, PTE was applied for the LFPs recorded from the Hp and NCL of pigeons during learning stage S3. The PTE was calculated 30 times for each pigeon. The results showed that the theta-band dPTE from the Hp to the NCL of the raw data was significantly higher than that of the surrogate data during the period from 0.2 s before T0 to 0.4 s after T0 for the pigeons in G1, and during the period from T0 to 0.6 s after T0 for the pigeons in G2 and G3, before the animals made a turn (Table 2, Figure 3c), suggesting that the information flowed preferentially from the Hp to the NCL during the visual–spatial associative task. The interaction lag values of pigeons were distributed in the range from 10 to 30 ms (Figure 3d). In summary, during the visual–spatial associative task, the theta-band oscillations in the Hp and NCL were strongly synchronized before the pigeons entered the critical choice platform to turn, with the information flowing preferentially from the Hp to the NCL. These findings indicate that the theta-band oscillations modulate the information interactions between the Hp and NCL in visual–spatial associative tasks.

### 3.3. The Dynamics of Hp–NCL Interaction during the Visual-Spatial Associative Learning

We then asked whether and how oscillatory interactions between the Hp and NCL evolved as pigeons learned to use visual cues to guide their navigational behavior. PLV was calculated for the trials in learning stages S1, S2, and S3. PTE was applied to each session, repeated 10 times. We focused on analyzing the interaction between the Hp and NCL during the period from ITI to the pigeon’s completion of behavioral response.

**Figure 4:**
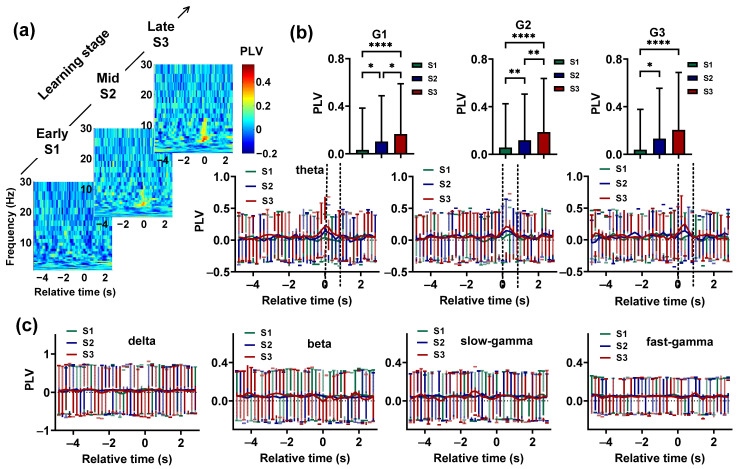
Evolution of the Hp and NCL synchronization during the visual–spatial associative learning task. (**a**) Example using pigeon P1 and the time-resolved Hp–NCL synchrony spectra for learning stages S1, S2, and S3. Time 0 (T0) indicates the end of the delay period, when the gate begins to open. (**b**) The theta-band synchronization between the Hp and NCL for the pigeons during learning stages S1, S2, and S3. The two dashed lines represent the beginning and full opening of the gate. Data are presented as mean ± std. Top: The theta-band *PLV* (calculated during the period from 0.4 s before T0 to 0.4 s after T0 for the pigeons in G1, the period from 0.2 s before T0 to 0.8 s after T0 for the pigeons in G2, and the period from T0 to 0.8 s after T0 for the pigeon in G3) of learning stages S1, S2, and S3. * *p* < 0.05, ** *p* < 0.01, **** *p* < 0.0001. Bottom: Theta-band *PLV* plotted as a function of the trial’s relative time for S1, S2, and S3. (**c**) The *PLV* of delta, beta, slow-gamma, and fast-gamma plotted as a function of the trial’s relative time for S1, S2, and S3. Data are presented as mean ± std.

We found that learning was accompanied by a steady increase in theta-band synchronization between the Hp and NCL (Figure 4a,b). To statistically analyze the effect of learning on *PLV*, the *PLV* of G1 during the period from 0.4 s before T0 to 0.4 s after T0, G2 during the period from 0.2 s before T0 to 0.8 s after T0, and G3 during the period from T0 to 0.8 s after T0 in learning stages S1, S2, and S3 were compared. The results showed that before the animals entered the choice platform, the Hp–NCL synchrony in S3 was stronger than in S1 and S2 (p < 0.0001, Chi-square = 15.2807 for G1; p < 0.0001, Chi-square = 19.0737 for G2; p < 0.0001, Chi-square = 14.3987 for G3; df = 2, Friedman ANOVA, detailed results of multiple post-hoc pairwise comparisons in Figure 4b). The synchronization of other frequency bands and task phases showed no change (Figure 4c). 

**Figure 5:**
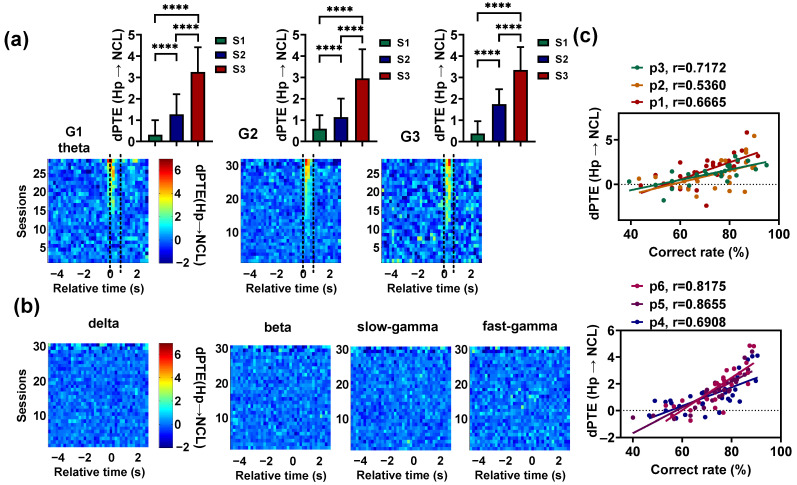
Dynamics of the Hp–NCL interaction during visual–spatial associative learning. (**a**) Top: The theta-band *dPTE* (Hp → NCL) (mean value during the period from 0.2 s before T0 to 0.4 s after T0 for the pigeons in G1, and the period from T0 to 0.6 s after T0 for the pigeons in G2 and G3) of the learning stages S1, S2, and S3. Time 0 (T0) indicates the end of the delay period, when the gate begins to open. Data are presented as mean ± std. **** *p* < 0.0001. Bottom: The theta-band mean *dPTE* (Hp → NCL) plotted as a spectrogram across the trial’s relative time and sessions. The two dashed lines represent the beginning and full opening of the gate. (**b**) The mean *dPTE* (Hp → NCL) of the delta, beta, slow-gamma, and fast-gamma plotted as a spectrogram across the trial’s relative time and sessions. (**c**) The correlations between the correct rate and the theta-band mean *dPTE* (Hp → NCL) for all pigeons. The *dPTE* (Hp → NCL) of the pigeons in G1 was calculated during the period from 0.2 s before T0 to 0.4 s after T0, and that of the pigeons in G2 and G3 was calculated during the period from T0 to 0. 6 s after T0.

We also found that the theta-band Hp–NCL interaction increased with learning before pigeons entered the choice platform (Figure 5a). To statistically analyze the effect of learning on  PTE, the mean dPTE of G1 during the period from 0.2 s before T0 to 0.4 s after T0, and the mean dPTE of G2 and G3 during the period from T0 to 0.6 s after T0 were calculated for each session. The theta-band dPTE (Hp → NCL) in S3 was obviously higher than the values in S1 and S2 (p < 0.0001, Chi-square = 228.3176 for G1; p < 0.0001, Chi-square = 352.1840 for G2; p < 0.0001, Chi-square = 142.8222 for G3; df = 2, Friedman ANOVA, detailed results of multiple post hoc pairwise comparisons in Figure 5a). However, for other frequency and task phases, no consistent significant information interaction was observed between Hp and NCL (Figure 5b). Finally, we calculated the correlation between the behavioral performance of pigeons and the theta-band mean dPTE (Hp → NCL) of each session. The mean dPTE (Hp → NCL) was calculated during the time period that showed significant interaction. The results showed a strong correlation (r = 0.6665, 0.5360, 0.7172, 0.6908, 0.8655, 0.8175 for P1–P6, respectively, p < 0.001 for P1; p < 0.01 for P2; p < 0.0001 for P3, P4, P5, and P6; Figure 5c). These outcomes suggest that visual–spatial associative learning is mainly associated with the increased interaction between the Hp and NCL at 4–12 Hz. 

### 3.4. The Enhanced Hp–NCL Interaction Predicts the Correct Behavior

To determine whether the coupling of 4–12 Hz oscillators in the Hp and NCL is necessary for a successful performance, we assessed the activity in learning stage S3 during incorrect trials (36 ± 11 trials for all pigeons). Due to the small number of incorrect trials in S3 and the accurate estimation of PLV requiring hundreds of trials [37], only PTE was applied for the incorrect trials (repeated 30 times). The analysis was focused on the period from ITI to a pigeon’s completion of behavioral response. The dPTE (Hp → NCL) of incorrect trials for each frequency band was compared with that of correct trials.

**Figure 6:**
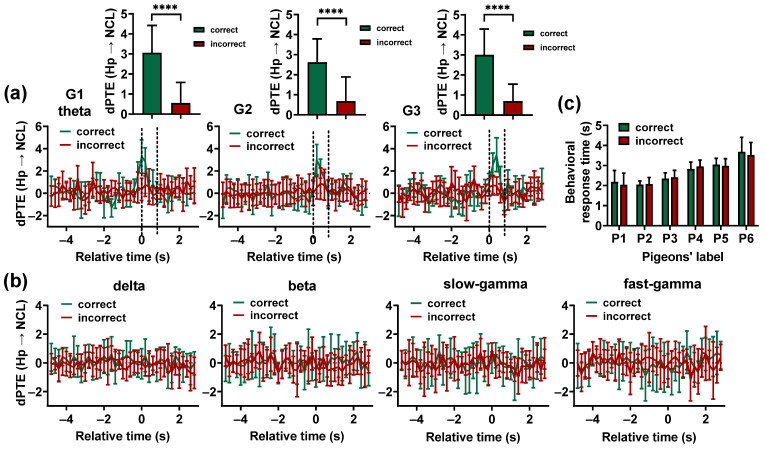
Comparing the results of the Hp–NCL interactions during correct and incorrect trials. (**a**) The theta-band *dPTE* (Hp → NCL) for the correct and incorrect trials plotted as a function of the trial’s relative time. Time 0 (T0) indicates the end of the delay period, when the gate begins to open. The two dashed lines represent the beginning and full opening of the gate. Data are presented as mean ± std. Top: The theta-band *dPTE* (Hp → NCL) (calculated during the period from 0.2 s before T0 to 0.4 s after T0 for the pigeons in G1, and the period from T0 to 0.6 s after T0 for the pigeons in G2and G3) of the correct trials was significantly higher than that of the incorrect trials. **** *p* < 0.0001. (**b**) The *dPTE* (Hp → NCL) of the delta, beta, slow-gamma, and fast-gamma frequency band plotted against the trial’s relative time. Data are presented as mean ± std. (**c**) Comparison of pigeons’ behavioral response times between correct and incorrect trials.

The results showed that no consistent Hp–NCL interaction was observed in incorrect trials for G1, G2, and G3, and the theta-band dPTE (Hp → NCL) before the animals entered the choice platform during the incorrect trials was significantly lower than during the correct trials (p < 0.0001, zval = 5.7935, sample size = 60 for G1; p < 0.0001, zval = 5. 9490, sample size = 90 for G2; p < 0.0001, zval = 4.1651, sample size = 30 for G3; Wilcoxon signed-rank test. Figure 6a). For other frequency bands and task phases, there was no significant difference between correct and incorrect trials (Figure 6b).

To establish whether the differences in the Hp–NCL interaction between correct and incorrect trials are caused by an animal’s speed of movement, a comparison was made between the behavior response time of pigeons in correct and incorrect trials. The results showed that there was no significant difference in the behavioral response time of pigeons between correct and incorrect trials (p > 0.05, zval = 0.3092, sample size = 40 for P1; p > 0.05, zval = 0.0217, sample size = 26 for P2; p > 0.05, zval = 1.6989, sample size = 25 for P3; p > 0.05, zval = 0.3375, sample size = 52 for P4; p > 0.05, zval = 1.8944, sample size = 42 for P5; p > 0.05, zval = 1.2980, sample size = 38 for P6; Wilcoxon signed-rank test. Figure 6c). However, the Hp–NCL interaction during correct trials was significantly larger than in incorrect trials, suggesting that the differences in the Hp–NCL interaction between correct and incorrect trials are not caused by the animal’s movement speed. These findings suggest that the 4–12 Hz interaction between the Hp and NCL before the animals make a choice is necessary for successful visual-based navigation.

## 4. Discussion

In this study, we investigated the interaction between the Hp and NCL of pigeons performing a visual–spatial associative learning task. The results showed that the theta-band (4–12 Hz) oscillations in the Hp and NCL became synchronized before the pigeons entered the critical choice platform to turn, and the Hp activity mainly drove that of NCL, with the information flowing preferentially from the Hp to the NCL during the visual–spatial associative task. The strength of the Hp–NCL interaction increased with learning, suggesting that visual–spatial associative learning is primarily associated with the increased interaction of 4–12 Hz rhythms. The enhanced Hp–NCL interaction predicted the correct choice, supporting animals’ use of visual cues for memory-guided decision making. These findings are consistent with our hypothesis and provide insight into the dynamics of the Hp and NCL coordination during visual–spatial associative learning, serving to reveal the mechanisms of the Hp–NCL network in spatial-related learning and memory.

We found that the Hp–NCL interaction in pigeons occurred at different moments of the task. For P1 and P2, a significant information interaction was observed during the period from 0.2 s before T0 to 0.4 s after T0, ahead of the pigeons in G2 and G3, whose Hp–NCL interaction occurred during the period from T0 to 0.6 s after T0. This may be related to the animal’s behavioral response speed. We repeatedly checked the video and found that the pigeons in G1 ran out in a hurry when the gate was opened enough for them to pass through, rushing to the choice platform, followed by the pigeons in G2. The pigeons in G3 had the slowest behavioral response speed and only started moving when the gate was fully opened. It seems that the pigeons need to complete the information interaction between Hp and NCL before turning. Therefore, due to the faster behavior response speed of the pigeons in G1, their Hp–NCL interaction occurs earlier than in other pigeons.

A large amount of evidence shows that the theta frequency band mediates the information processing and communication between the Hp and PFC in mammals [43,44,45]. Studies on rodents have suggested that the theta-band mediates the spatial-related information interaction between the Hp and PFC in spatial-related tasks [6,46,47]. The theta-band also mediates other information interactions. In a study about rats performing an odor–spatial associative task, the researchers found that upon context entry, the spatial context information was communicated from the Hp to the PFC through the theta-band. However, upon the onset of odor sampling, the direction of information flow reversed, with the PFC exerting a top–down control signal over the Hp via the theta-band to dominate the memory retrieval [48]. This pattern of theta-band modulation of the information interaction between the Hp and NCL has also been reported in pigeons. When pigeons performed a spatial learning task, the connection between the Hp and NCL gradually increased in the theta-band, accompanied by the formation of a stable routing [28]. In this study, we found a theta-band interaction between the Hp and NCL of pigeons performing a visual–spatial associative task, consistent with the results of rats performing an odor–spatial associative task. When the rats left the odor sampling port and started running towards the reward site, the significant synchronization between the Hp and PFC in the theta-band was detected, which was considered as the theta-driven spatially modulated activity supporting spatial navigation for animals [12]. Therefore, the theta frequency band might be the critical band modulating the information interaction between the Hp and NCL in avians. 

There are some limitations in the present study. Firstly, what information do the bird Hp and NCL interact with during visual–spatial associative learning? In mammals, it is thought that the spatial associative information is initially encoded in the Hp and then transferred to the neocortex to store and integrate within pre-existing memory traces during slow-wave sleep (SWS) [49,50]. During associative memory retrieval, the contextual information is passed from the Hp to the PFC to support this procedure [12,13]. Meanwhile, the PFC exerts control signals over the Hp to avoid the re-encoding of remote information in the Hp. However, despite pathological studies indicating that bird Hp plays an important role in visual–spatial associative learning and some researchers suggesting that spatial associative information may be encoded in bird Hp, unlike mammals, SWS and rapid eye movement sleep related to memory transfer have not yet been found in the bird brain [50]. So, where is the associative memory stored? One assumption is that it is stored in Hp, and the sleep-related synaptic down-scaling rectifies the saturation in Hp. So, the Hp–NCL interaction may be the spatial associative memory communication from the Hp to the NCL that supports decision making. Another hypothesis is that the birds may have evolved an as-yet unidentified mechanism to store their spatial associative memory, and the bird Hp and NCL share visual information to support the associative memory recall. It might also be that the spatial-related information modulated by the theta-band is communicated between the Hp and NCL, like that in the odor–spatial associative task performed by rats.

Secondly, although there are multiple indirect connection routes between the bird Hp and NCL, which brain region serves as the intermediate region in visual–spatial associative learning to modulate their connections? The graph–theoretical analysis presented in [33] revealed a connective core of five inter-connected hub nodes in a pigeon’s forebrain, including the arcopallium dorsale (AD), arcopallium intermedium (AI), area corticoidea dorsolateralis (CDL), APH, and NCL. In graph–theoretical terms, they are the most topologically central regions and the most richly connected to the rest of the network, thus serving as the central information flow in the bird brain. In these hub modes, AD, AI, CDL, and APH show bidirectional connections with Hp, which may provide indirect pathways between the Hp and NCL. However, so far, it is unknown which brain region modulates the information interaction between the Hp and NCL, serving as the intermediate region.

Thirdly, to eliminate the differences in the Hp–NCL interaction between correct and incorrect trials caused by animal movement speed, we compared the behavioral response time of pigeons between correct and incorrect trials, which is inversely proportional to the average speed, as the displacement from the home area to the target arm is a constant. Ideally, the relationship between PTE and momentary speed should be established. However, due to the limitations of the experimental equipment, the momentary speed of animals cannot be detected. In future research, more rigorous methods are needed to address this issue, such as monitoring the animal’s head orientation and the momentary speed of animals.

Finally, as shown in Figure 3b and Figure 4, the standard deviation of PLV was very large. The reason for this is the different distances between the channels in the microelectrode array and the task-response neurons; some channel pairs showed a significant increase in PLV, while others remained at a low level, resulting in a large standard deviation for PLV. In this paper, another method, ensemble PTE, was applied to measure the interaction between the Hp and NCL of pigeons during the visual–spatial associative learning task, and this was proven to be an effective method for identifying information interaction between brain regions [51]. Theoretically, the ensemble PTE is suitable for trials with the same neural activity, but not applicable for the learning process, during which the neural activity changes rapidly [52]. However, in our experiment, the pigeons needed about 28 days to complete the learning process, and the learning process was very slow, so we assume that the pigeon brain has the same neural activity in a session. In our report, the same results were obtained with the ensemble PTE and PLV, which indicates that the assumption seems to be valid. We look forward to more research focusing on the application of the ensemble PTE in the neural signals during slow learning processes.

## 5. Conclusions

In this paper, to investigate the interaction between the Hp and NCL of pigeons during visual–spatial associative learning, we monitored neural activity of the Hp and NCL in pigeons performing a visual–spatial associative learning task, and the phase locking value and phase transfer entropy were applied to local field potentials of the Hp and NCL. We found that the theta-band oscillations in the Hp and NCL were highly synchronized before the pigeons entered the choice platform to turn, and the Hp activity was the main driver of the theta-band oscillations of the NCL, indicating that the information flowed preferentially from the Hp to the NCL. The interaction strength of the Hp–NCL network increased with learning, suggesting that the learning process was associated with increased theta-band interactions. The enhanced theta interaction between the Hp and NCL is associated with successful choice, supporting the idea that animals use visual cues to guide their navigational behavior. These results provide insight into the Hp–NCL interaction of pigeons during visual–spatial associative learning and can serve to reveal the mechanisms of the Hp–NCL network in learning and memory.

## Figures and Tables

**Table 1:** Statistical results of theta-band *PLV* for G1, G2, and G3 at different task time bins with paired *t*-test. * *p* < 0.05; **** *p* < 0.0001; n.s.: *p* > 0.05. Time 0 indicates the end of the delay period, when the gate begins to open.

Groups	−0.4–0 s	−0.2–0.2 s	0–0.4 s	0.2–0.6 s	0.4–0.8 s	*df*
	*p*/tstat	*p*/tstat	*p*/tstat	*p*/tstat	*p*/tstat	
G1	****/3.8765	****/4.9466	****/4.0944	n.s./1.9217	n.s./0.5177	480
G2	n.s./1.8414	****/4.2550	****/6.3351	****/5.8486	****/3.9008	705
G3	n.s./0.0355	n.s./1.1855	****/3.4135	****/4.3204	*/2.3957	255

**Table 2:** Statistical results of theta-band *PTE* for G1, G2, and G3 at different task time bins with binomial test. * *p* < 0.05; ** *p* < 0.01; *** *p* < 0.001; **** *p* < 0.0001; n.s.: *p* > 0.05. Time 0 indicates the end of the delay period, when the gate begins to open.

Group	−0.4–0 s	−0.2–0.2 s	0–0.4 s	0.2–0.6 s	0.4–0.8 s	Sample Size
G1	n.s.	***	**	n.s.	n.s.	60
G2	n.s.	n.s.	****	**	n.s.	90
G3	n.s.	n.s.	*	**	n.s.	30

## Data Availability

Data are available upon request to the corresponding author.

## Supplementary Materials

The following supporting information can be downloaded at: https://www.mdpi.com/article/10.3390/ani14030456/s1, Table S1: behavioral data of pigeons during visual–spatial associative learning; Figure S1: Theta-band dPTE  (Hp → NCL) with different bin sizes; Table S2: Statistical results of theta-band PTE with different bin sizes; Figure S2: the theta-band PLV for P1–P6; Table S3: Statistical results of theta-band PLV for P1–P6; Video S1: the visual–spatial associative learning of pigeons in the “Y” maze. Video S1 can be downloaded at https://zenodo.org/records/10075081.
