# Supplementary material for: Enhanced Hippocampus–Nidopallium Caudolaterale Interaction in Visual–Spatial Associative Learning of Pigeons
Source: Animals (Basel). 2024 Jan 30;14(3):456. doi: 10.3390/ani14030456 (PMC10854635; doi:10.3390/ani14030456)
Supplement: Supplementary file 1 [file animals-14-00456-s001.zip › animals-2731447-supplementary.pdf]

Supplementary materials

Table S1. Behavioral data of pigeons in visual–spatial associative learning tasks.

Session	P1	P2	P3	P4	P5	P6
	**Correct/ All trials**	**Correct/ All trials**	**Correct/ All trials**	**Correct/ All trials**	**Correct/ All trials**	**Correct/ All trials**
1	17/32	18/29	17/32	17/31	16/40	19/34
2	20/33	22/36	16/32	16/26	18/37	21/35
3	18/27	22/37	16/41	16/33	20/35	19/36
4	20/35	17/39	18/32	19/36	20/32	20/36
5	17/25	23/35	21/33	17/36	17/25	22/35
6	21/42	20/35	20/28	20/29	21/35	20/30
7	18/25	22/33	22/39	21/37	24/34	22/35
8	19/43	21/26	21/37	20/34	22/35	24/33
9	21/35	22/35	17/26	23/34	24/33	23/30
10	19/29	25/31	19/25	19/34	24/34	22/30
11	22/35	16/32	18/22	20/29	22/30	21/30
12	22/33	18/24	17/24	15/28	17/22	23/30
13	16/24	19/22	19/30	22/29	19/25	20/27
14	17/28	18/23	23/29	20/29	18/26	18/24
15	20/27	20/26	17/22	23/30	20/27	19/25
16	21/30	20/25	22/30	21/27	20/27	22/30
17	26/37	18/21	21/27	16/25	21/27	23/30
18	20/27	19/23	16/20	20/29	22/28	17/22
19	21/28	20/22	18/23	23/30	18/23	25/31
20	25/31	19/25	20/24	24/32	21/27	20/26
21	22/29	21/27	20/25	23/29	17/21	24/29
22	19/23	19/22	21/25	24/29	24/30	23/28
23	20/24	20/22	26/30	18/23	22/27	22/25
24	21/26	25/29	20/22	15/19	24/30	24/28
25	21/26	20/23	25/26	19/23	19/22	22/27
26	25/29	22/24		23/26	26/30	26/30
27	24/26			17/21	27/32	22/25
28	20/23			21/25	24/27	21/24
29				22/24		
30				26/30		
31				23/26		

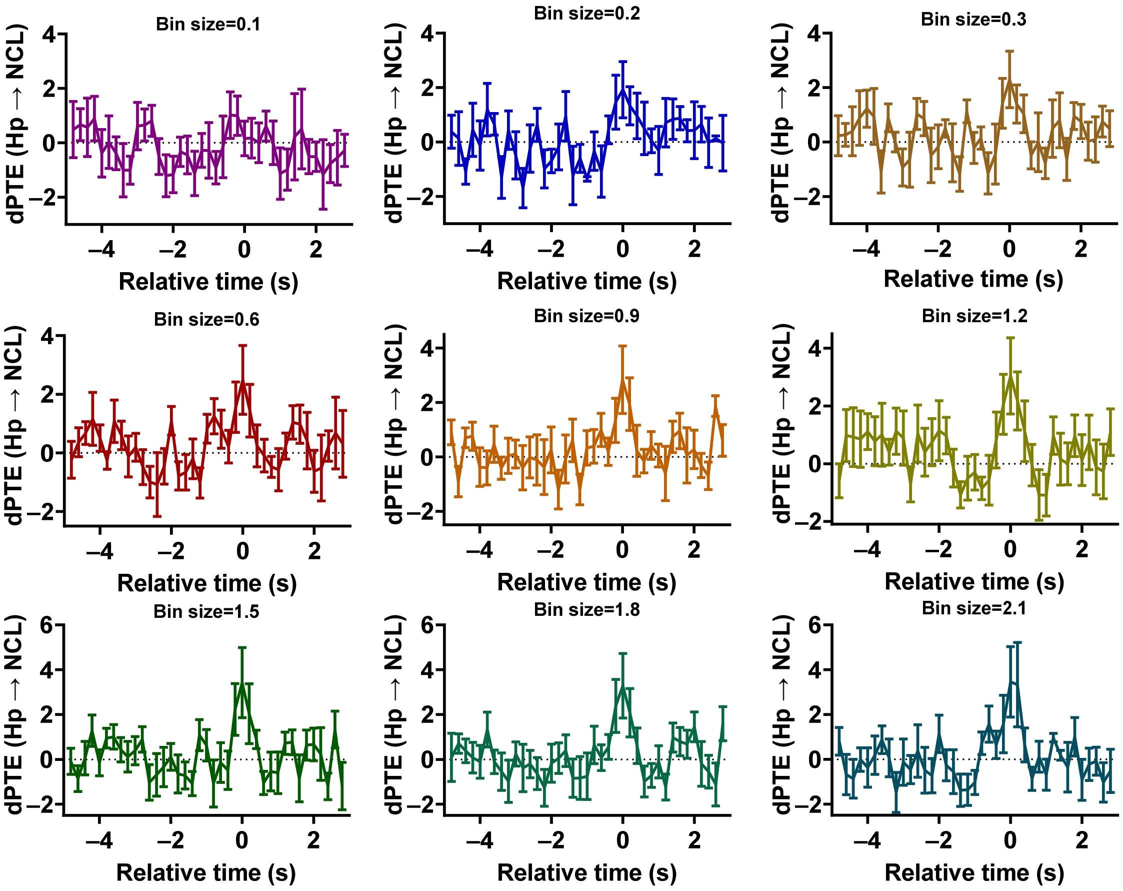

Figure S1. Theta-band $dPTE$ (Hp → NCL) with different bin sizes. Time 0 (T0) indicates the end of the delay period, when the gate begins to open. The P1 pigeon was as an example to analyze the impact of bin size on the results. The PTE was applied to the LFP in learning stage S3 of P1, and the analysis was focused on the period from ITI to the pigeon's completion of behavioral response. The bin size increased from 0.1 to 2.1, and for each bin size, the $dPTE$ was calculated 30 times. The significance of the $dPTE$ value for each estimation was tested against the 200 surrogate data sets ($p < 0.05$), and then the binomial test was applied to establish the statistical significance over all repetitions. The statistical results are presented in Table S2.

Table S2. Statistical results of the theta-band PTE with different bin sizes. *$p < 0.05$; **$p < 0.01$; ***$p < 0.001$; n.s.: $p > 0.05$.. Time 0 indicates the end of the delay period, when the gate begins to open.

	**−0.4–0 s**	**−0.2–0.2 s**	**0–0.4 s**	**Sample size**
Bin size = 0.1	n.s.	n.s.	n.s.	30
Bin size = 0.2	n.s.	n.s.	n.s.	30
Bin size = 0.3	n.s.	*	n.s.	30
Bin size = 0.6	n.s.	*	n.s.	30
Bin size = 0.9	n.s.	*	*	30
Bin size = 1.2	*	*	*	30
Bin size = 1.5	*	**	*	30
Bin size = 1.8	*	**	*	30
Bin size = 2.1	n.s.	**	**	30

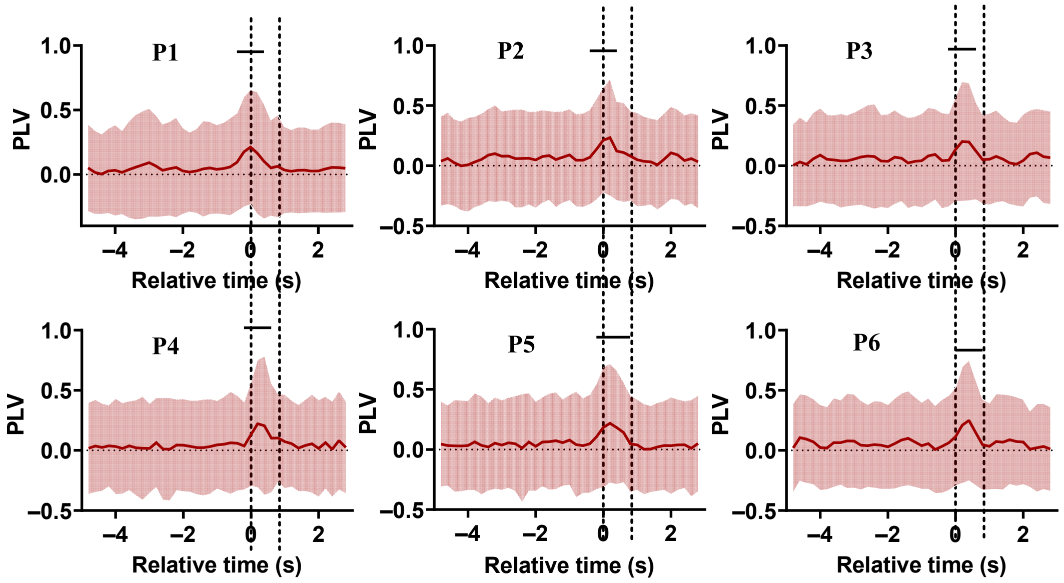

Figure S2. The theta-band *PLV* for P1–P6. Time 0 (T0) indicates the end of the delay period, when the gate begins to open. The two dashed lines represent the beginning and full opening of the gate, respectively. "—" indicates the significant difference. Data are presented as mean ± std.

Table S3. Statistical results of theta-band *PLV* for P1–P6 at different task time bins. *$p < 0.05$; **$p < 0.01$; ***$p < 0.001$; ****$p < 0.0001$; n.s.: $p > 0.05$. Time 0 indicates the end of the delay period, when the gate begins to open.

Pigeons ' label	−0.4–0 s	−0.2–0.2 s	0–0.4 s	0.2–0.6 s	0.4–0.8 s	*df*
	p/ tstat	*p*/ tstat	*p*/ tstat	*p*/ tstat	*p*/ tstat	
P1	**/2.7589	****/3.7141	**/2.6676	n.s./1.3831	n.s./0.1310	224
P2	**/2.7227	**/3.2939	**/3.1120	n.s./1.3318	n.s./0.8486	255
P3	n.s./0.7417	**/2.7687	****/3.4962	**/3.2584	*/2.4118	255
P4	n.s./0.9477	*/2.3624	**/3.0093	**/2.6805	n.s./1.1111	224
P5	n.s./1.8998	**/3.1174	****/3.8556	**/3.1235	*/2.4509	224
P6	n.s./0.0355	n.s./1.1855	****/3.4135	****/4.3204	*/2.3594	255
